# Surgery

**Published:** 1837-07

**Authors:** 


### SURGERY.

*Case of Popliteal Aneurism.* By R. Middlemore, Esq. Birmingham.

This case presents no peculiarities, except that rupture of the aneurismal sac took place from a fall about six weeks after the operation, and without any subsequent ill consequences. We notice it chiefly for the purpose of introducing the following remark by Mr. Middlemore.

"It appears to be a great advantage to cut through the skin and adipose membrane quite down to the fascia, or as nearly so as possible, with one sweep of the knife; for it may be remarked that when the skin and fat are divided by separate incisions, the wound is by no means so wide—does not gape so freely, and therefore does not afford so much room for subsequent manipulations—as when these textures are conjointly severed. There is a difficulty in making the necessary calculations, so as to apply the degree of force required to divide the skin and adipose substance merely, which none but an experienced operator can readily surmount. I am satisfied that an inability to accomplish this, or an unacquaintance with the necessity of doing so, occasions much delay in the performance of many surgical operations." *British Annals of Medicine. March* 10, 1837.

---

*Compound Luxation of the Humerus.* By P. T. Scott, Esq.

A lad, æt. fourteen, had his shoulder dislocated by a fall from a horse, and the head of the bone thrust so far from its natural position as to be found "lying exposed on the anterior part of the chest, over the pectoral muscle." There was considerable loss of blood, and the integuments were greatly lacerated, and the first aspect of the wound was such as to suggest the propriety of amputation.

However, the limb was reduced, and the cure was affected without any untoward symptom; and the limb is now, in every respect, sound and supple.

*Lancet. March* 4, 1837.

---

*On the Operation for Hernia, without dividing the Sac.*
By M. W. Hilles, Esq.

To those who are acquainted with the records of surgery, or who have read Mr. Key's valuable memoir on this subject, Mr. Hillis's communication will possess no novelty. It is singular that he should consider the proposal made by him as new. This he does, he says, because he thinks no one before him proposed the division of the stricture outside the sac, "with the specific object of facilitating the reduction of the hernia by the taxis." To show Mr. H. how surgeons in general think on this point, we quote a sentence from our review of Dr. Parrish's work in our last Number. "He seems hardly to be aware of the object and intention which the surgeon has in view by dividing the stricture external to the sac; namely, that he may employ the taxis under a condition of parts which he has rendered more favorable to its successful application, in preference to subjecting the patient to the risk of peritoneal inflammation by opening the abdominal cavity." We ourselves, however, think so favorably of the plan of operating outside the sac, that we are not sorry that Mr. Hilles has brought the subject forward in the journals. Our readers are referred for a full account of it to Mr. Key's work.

*Lancet. February* 11, *April* 15, 1837.

---

*Cases and Communications illustrative of Subjects in Military Surgery.*
By Sir George Ballingall, Professor of Military Surgery.

These are cases communicated to Sir George, by his pupils and friends. The following are outlines of them:

*First Case,* (by A. S. Allen, M.D., Surgeon, R.N.) An Arab received a sabre wound, which traversed the heads of the metacarpal bones of the little ring, and middle finger of one hand, and the same bones of the opposite hand about an inch nearer their digital extremities, dividing at the same time all the arteries, veins, nerves, &c. in its course. The hemorrhage was considerable, but was ultimately restrained by tight bandaging and styptics. Dr. Allen saw the patient, for the second time, three weeks after the accident, when reunion of all the bones had taken place.

*Second Case*, (by the same.) *Spontaneous Separation of the Leg at the Knee-joint, from Gangrene.* Six months after the accident, Dr. Allen amputated the limb, sawing through the bone about an inch above its middle. Two small arteries, branches of the profunda, required ligatures; but, in the situation of the femoral artery, nothing could be observed but a small mass of firmly condensed cellular membrane.

*Third Case,* (by J. STEVENSON, Esq., Surgeon, E. I. C. S.) An Indian received a sabre wound a little below the outer edge of the deltoid, which cut across the belly of this muscle, and also the os humeri. The hemorrhage was arrested by tying a turban round the arm with great tightness. Amputation not being allowed, an attempt was made to heal the wound, which completely succeeded. No hemorrhage supervened, and the pulse could be perceived at the wrist on the third day. The wound was nearly all cicatrized on the twenty-sixth day; and on the forty-fifth the bone was firmly united. It is stated that, on the thirteenth day, the pulse was sixty-eight in the wounded arm, and eighty-two in the sound: was this really the case?

*Fourth Case,* (by MR. STRATTON.) *Fifth Case,* (by the late DR. MACKENZIE.) These were both examples of the division of the bone of the thumb by sharp instruments, and complete reunion by the first intention. In Dr. Mackenzie's case, the patient brought the separated portion of his thumb in his pocket.

*Edinburgh Journal. April,* 1837.

---

### *Observations on Extraction and Displacement of the Cataract.*

By J. A. ROBERTSON, M.D. Surgeon to the Eye Dispensary of Edinburgh.

THIS is a very excellent statistical and practical paper, and, as far as it goes, places in a clear light the relative success resulting from the different modes of performing the operation for cataract. Dr. R. passes in review the advantages and disadvantages of—1. *Division,* or breaking down the lens in situ; 2. *Extraction;* 3. *Displacement;* including *Depression* and *Reclination;* in the former process, the lens being pushed perpendicularly downwards; in the latter, the lens being imbedded in the vitreous humour, with its anterior surface turned upwards and its upper edge backwards. We can only find room to give some of the numerical results deduced by Dr. R. from the collation of numerous cases, and the conclusions which he has found himself warranted to draw from the whole facts and reasonings.

Out of 1307 operations by *Extraction,* there were 397 failures, or 30 per cent. (nearly). Out of 5729 operations by *Displacement,* there were 1004 failures, or 17 per cent. (nearly). This last series includes a great number of operations by *Depression* as well as *Reclination;* and Dr. R. remarks, that inasmuch as the latter operation is preferable to the former, so ought the results to be much more favorable if all the cases had been operated on by Reclination. The preceding results are collected from the writings of others. Dr. Robertson subjoins a statement of 179 cases of hard cataract, without any complication, operated on by himself. The results were as follows:

| | Cases. | Cured. | Relieved. | Failed. |
|---|---|---|---|---|
| Extraction, ...... | 64 | 32 | 14 | 18 |
| Reclination, ......... | 115 | 94 | 10 | 11; |

being a proportion of failures by Extraction of 28 per cent. (nearly), and by Reclination of only 9 per cent. (nearly).

The following are the conclusions drawn by Dr. R.:

"1. That the safest mode of operating when the cataract is soft is by breaking it down, and thereby effecting its absorption.

"2. That in all operations in which the needle is used, that instrument ought to be entered through the sclerotic, and not through the cornea.

"3. That in cases of hard cataract, complicated with the various diseases of the eye and its appendages, mentioned above, the needle ought to be employed, the mode of operating being adapted to each individual case.

"4. That hard cataract without such complications may be effectually removed by the operations of *Extraction* or *Displacement*.

"5. That the operation of *Depression* is impracticable, and that attempts to perform it will be productive of the most injurious results.

"6. That many of the accidents which may occur during the operation of *Extraction* cannot be avoided by any degree of skill or dexterity, and, therefore, form valid objections to the operation.

"7. That the accidents which may occur during the operation of reclination arise from faults on the part of the operator, for as soon as the needle is entered, he has complete command over the eye. They do not, therefore, form valid objections to the operation.

"8. That the resulting success from the operation of *Reclination* is very much greater than that from *Extraction*.

"9. That in cases of failure of the operation by *Reclination*, the eye, in the majority of instances, is not left in so hopeless a condition as when the operation of *Extraction* has failed.

"10. That, in consequence of the comparatively rare occurrence of violent inflammation after *Reclination*, there is infinitely less likelihood, than in cases of *Extraction*, of such depleting measures being required as may prove ruinous to the general health of the patient." *Edinburgh Journal. April*, 1837.

---

### *On the Application of solid Nitras Argenti in the Gonorrhœa of Women.* By A. J. Hannay, M.D. Glasgow.

This is a valuable practical paper, and well worthy the attention of surgeons who are called on to treat the class of cases to which it refers. The following extracts give the essential parts of Dr. H.'s communication.

"Having noticed that some cases of gonorrhœa, with ulceration in the vagina, and to which I had freely applied the nitras argenti, stopped in a most extraordinarily short period, I determined to select a few cases of uncomplicated gonorrhœa for trial. I accordingly did so, and found the effects to be very striking. I have repeatedly seen the discharge cease, never to return, in twenty-four hours. On the day after the application, I have often seen it changed in character, that is, lose its purulent form, and disappear in twenty-four hours more. I have kept such cases repeatedly under my eye for a month, and can declare that in the proportion of 95 in 100 there was no return of the discharge; yet no remedy had been employed after the nitrate. Though in some few cases there is a little pain produced, yet in by far the greatest number of instances no pain is experienced from the introduction and most free application of the caustic. It is painful, it is true, when it touches sores on the labia or more internal parts, but that smarting soon goes off, or, at all events, an anodyne instantly relieves it. In the greater number of cases it produces no phlogosis of the parts with which it comes in contact; but in some (say in one case in twenty) it does irritate to a degree that proves painful for a few hours, but never in any one instance have I seen the pain continue longer than a few hours. I have never seen bubo induced by it. I have used it in patients in every month of pregnancy with the best effects, and never saw abortion produced. I know, by as careful examination as I could make, that it does not suppress the catamenia: in short, I fearlessly give it out as an infallible and safe remedy for this disease, without any one drawback but the vain fears of persons of no experience, or of such as are determined to oppose it. I have now employed it in above three hundred cases with unvarying success." . . . "I may add, that in about six cases only, out of more than three hundred, the vaginal discharge has continued after repeated applications: in all of these I found, by the use of the speculum vaginæ, that there was ulceration of the lining membrane of the vagina, and that the case was not gonorrhœa. The application of the nitrate was not required more than once in 280 of the 300 cases."

The following is Dr. H.'s account of his mode of using the remedy.

"I introduce a stick of nitrate of silver into a quill, and tie a thread firmly round the lower part of the quill to fasten the caustic, which I leave projecting beyond the quill about half an inch. I generally smear the quill with a little lard, and introduce the nitras argenti up to the os tincæ, or as far as it can be made to ascend in the vagina. I then deliberately and slowly withdraw it, turning it round so as to bring it in as extensive contact as possible with the lining membrane of the vagina. I may add, that by accident the nitrate of silver has more than once broken in the vagina, and could not be found. It caused me much alarm and anxiety at first, but though I would carefully avoid it, I now regard the occurrence as of very little importance." *Med. Gazette. May* 6, 1837.

[Since the paper of which the foregoing is an abstract was published, the following important criticism on it has been published by Dr. J. M'Cune Smith, who was clerk to the Glasgow Lock Hospital, the institution in which Dr. Hannay's observations were made.]

"1st. I deem the treatment of gonorrhœa in the female by the solid nitrate of silver a cruel practice, from the horror which those who have undergone it express at what they call the 'burning their inside with caustic;' and also from a fact, of which the journals of the house give ample evidence, that the Lock Hospital is always full whilst Dr. Cumin has charge of it, but, on the other hand, does not average above four-fifths full whilst under the charge of Dr. Hannay.

"2dly. I deem the practice inefficient, because, of five patients who remained in the house on the 1st of January, 1837, '*cured*,' according to the statement in the journal, by means of 'the solid nitrate of silver,' the discharge returned in all of them except one within a fortnight afterwards, and that individual was dismissed on the 7th of January. Of the five patients above alluded to, in one the catamenia have not appeared since (whilst there is no sign of pregnancy,) after a lapse of four months. A second, shortly after the application, was seized with a sanguineous discharge from the uterus, which, after lasting fifteen days, terminated in an early abortion. Another patient (B. K., page 341) stated, that on the very day of the application of the nitrate of silver, in the agony caused by it, she so strained the abdominal muscles as to cause double inguinal rupture, which she now labours under." *Med. Gazette. May* 27, 1837.

---

*Spontaneous Mortifications of the Toes treated by tightly Bandaging the Leg.*
By J. C. SPENDER, Esq., Bath.

THIS case illustrates very happily the tonic influence of bandaging in a relaxed condition of the soft parts. When first seen by Mr. S., there was already mortification of one toe and incipient gangrene of the others. The following extract shows the condition of the parts and the progress of the cure:

"On examining the leg it was found to be considerably swollen, from the presence of adventitious deposits of a fluid and yielding character, particularly along the dorsum of the foot, and up the sides of the limb. Supposing that this unhealthy condition of the superficial structures of the leg materially contributed to increase, even if it did not produce that inactivity of the circulation, &c. upon which the mischief in the toe would be seriously augmented, I immediately applied a flannel roller very tightly around the limb from the root of the toes up to the knee, first covering the great toe with a simple dressing, and enveloping the second in an additional piece of flannel. The patient passed a better night, and on the next day the bulkiness of the limb was diminished, whilst the inflammation, which before had been extending along the dorsum of the foot, was arrested. The patient was directed to take half a pint of decoction of bark during the day, and the same quantity of wine, together with a full supply of meat. The bandage was again tightly applied, and over it a second, for the double purpose of increasing the warmth of the limb and securing the full effects of compression. Under these measures there was a daily improvement, so that at the end of a week the leg was reduced to its proper size, the inflammation on the upper surface of the foot had disappeared, the second toe was restored to its natural temperature, and nearly to its natural colour, whilst the curative stages were proceeding most favorably and quickly in the great toe itself. A line of demarcation was forming around the toe between the dead and living parts immediately contiguous to the posterior boundary of the original slough, but extending rather farther back at the surface of the toe opposed to the second. At this time the nail becoming loose, and finding it in the way, I thought it would be better to hasten its removal, together with its attached cuticle, by applying a linseed poultice; and the bandage was omitted, from being unable to commence its application from the root of the toes, in consequence of the presence of the poultice. After the poultice had been employed for two days, there was a frightful return of the old symptoms. The inflammatory state of the dorsum of the foot reappeared, the second toe was again livid, the limb was increased in bulk. I immediately threw away the poultice, applied very tightly the two bandages, and was glad to find that on the next day there was an arrest, and on the day after a removal of all the unfavorable symptoms. The bandaging was steadily pursued, the line of demarcation in the great toe progressively deepened, and the dead parts of the toe, embracing the first phalanx, and all the soft structures covering it, daily became looser and looser."

The recovery was complete, with the loss of the great toe.

*Med. Gazette. May* 13, 1837.

---

*Case of Laceration of the Diaphragm.* By T. B. Curling, Esq. Assistant Surgeon to the London Hospital.

Cases of this kind, whether from injuries or spontaneously, are happily rare: only two instances (both of the first form,) have occurred at the London Hospital during the last ten years, where the number of accidents annually admitted considerably exceeds a thousand. The case now described is that of a strong muscular man, who fell from a great height, and was brought to the hospital with fracture of the arm, ribs, &c. He lived some days, during which time he experienced considerable dyspnœa and pain at the sternum; and it was observed that he breathed only by the right lung. On inspection after death, the eight superior ribs on the left side were found fractured; the left lung was completely collapsed, and pushed up to the top of the chest; and there was a rent, four or five inches long, in the diaphragm, through which the stomach, omentum, spleen, half the duodenum, the transverse colon, and a part of the thin edge of the left lobe of the liver, had passed into the left cavity of the pleura.

*British Annals of Medicine. May* 5, 1837.

